# Nutritional Profiling and Antioxidant Property of Three Wild Edible Mushrooms from North East India

**DOI:** 10.3390/molecules27175423

**Published:** 2022-08-25

**Authors:** Joshua Khumlianlal, K. Chandradev Sharma, Leichombam Mohindro Singh, Pulok K. Mukherjee, Sarangthem Indira

**Affiliations:** 1 Microbial Resources Division, Institute of Bioresources & Sustainable Development (IBSD), An Autonomous Research Institute of Department of Biotechnology, Government of India, Takyelpat, Imphal 795001, Manipur, India; 2 School of Biotechnology, Kalinga Institute of Industrial Technology, Bhubaneswar 751024, Odisha, India

**Keywords:** edible mushrooms, minerals, vitamins, HPLC analysis, phenolic compounds, organic acids, antioxidants

## Abstract

The mushroom is an important food for the rural tribal populations in Manipur, because of its high nutritional contents. In this study, we report on the nutritional profile of three wild edible mushrooms consumed by the tribal populations of Manipur viz.: *Macrocybe gigantea* J124; *Lactifluus leptomerus* J201 and *Ramaria thindii* J470. The studied mushrooms possess a high protein content of 37.6%, 20.8% and 16.4%, respectively. They have a high vitamin C content with low vitamin B1, B2 and folic acid. Among the three mushrooms, *M. gigantea* J124 possesses the highest mineral content, followed by *R. thindii* J470 and *L. leptomerus* J201. The total phenolic content of *L. leptomerus* J201, *M. gigantea* J124 and *R. thindii* J470 were 26.206, 29.23 and 30.99 mg GAE/g, with flavonoid content of 6.646, 6.854 and 9.187 mg quercetin/g, respectively. *R. thindii* J470 has the highest TPC and TFC content, which correlates with its DPPH radical scavenging activity. The IC50 values for *R. thindii* J470, *M. gigantea* J124 and *L. leptomerus* J201 are 242.0 µg/mL, 550.4 µg/mL and 689.0 µg/mL, respectively, which suggest that the higher content of phenolic compounds in *R. thindii* J470 contributes to its radical scavenging properties.

## 1. Introduction

Manipur is a hill-girt state with an oval shape valley at the center, which is surrounded by nine ranges of hills on all sides. Geographically in the north-eastern region of India, covering a total area of 22,327 sq. km, lying between 23°50′ and 25°41′ North latitudes and 93°61′ and 94°47′ East longitudes [1]. The region falls in the Indo-Burma Global Biodiversity hotspots, experiencing different types of climatic zones, ranging from tropical to sub-tropical and temperate forests [2]. This region is also known for its high humidity during the monsoon period, providing ideal agro-climatic conditions for the growth of different species of mushrooms [3]. Reports regarding the nutritional values of edible wild mushrooms have been reported in different states of Northeast India, including Tripura, Assam, Arunachal Pradesh, Meghalaya and Nagaland [4,5,6,7,8,9,10,11,12,13], but there are only a few reports available from Manipur [9,10,13]. Mushroom gathering is an important income-generating activity and a source of food for the rural tribes of Manipur, as the hill districts have a rich diversity of edible mushrooms.

Since time immemorial, wild edible mushrooms have been consumed by man as an important source of nutrition and medicines, due to their well-established therapeutic potential [14]. It has been reported that many wild edible mushrooms contain an exorbitant amount of proteins, vitamins and other major and minor minerals, such as magnesium, calcium, sodium, iron, zinc, selenium, etc., and low calorific values [15,16,17]. The fruiting bodies and mycelium of some of the species of mushrooms are reported to contain important bioactive compounds with a high antioxidant potential [17]. Besides the high nutritional value, they have a well-known medicinal utility due to their well-established therapeutic potential [18,19]. Owing to their beneficial effects on human health, edible mushrooms have become increasingly popular as functional foods [20]. Ever since, with the increase in the consumption of mushrooms for their nutritional benefits, data on their nutritional values are needed to ensure the safety of their consumption by the public [21].

*Macrocybe gigantea* [22] is an edible mushroom which is of important nutritional, medicinal and economic value. It has been reported that *M. gigantea* tastes sweet and is rich in nutrient constituents, such as protein, polysaccharides, fat, amino acid and many mineral elements [23]. In addition to its palatability, it has been reported that it possesses certain medicinal values, such as augmented antioxidant properties, anti-bacterial and anti-tumor effects [22,24].

Some species of the genus *Ramaria* have been reported as being consumed for their nutritional purposes [25]. They can be recognized by their fleshy, thickly branched fruiting bodies and tough textures, or sometimes partly gelatinous or jelly-like structures [25]. They are reported to possess antitumor activity and to show augmented antioxidant defense activities [26], and their ethanolic extract is also known to possess DPPH free radical scavenging and anti-lipid peroxidation qualities attributed to flavonoids and phenolic compounds [27].

*Lactifluus,* belonging to the family *Russulaceae*, is the most commonly reported wild mushroom variety consumed by the tribal community in Manipur. Most of *Russula* are an excellent source of digestible fiber, protein, vitamins and minerals [28]. Owing to its flavor, texture and excellent taste, the edible species of *Russula* is popular in Manipur. The sesquiterpenoids contribute to the unique color of *L. deliciosus*, some of which are known to possess potent biological activities, such as antimicrobial and anticancer [29,30,31]. Its extracts also have various biological activities, such as the inhibition of enzymes, antioxidant, antimicrobial and anti-inflammatory effects [31,32,33]. However, more studies are required in order to detect the nutraceutical and nutritional content of these wild edible mushrooms.

The rural tribal populations of Manipur consume the wild, edible mushrooms growing in the forest beds. Proper scientific studies on the nutritional profiles of the mushrooms from northeast India are scarce. Hence, in the present study, we analyzed the proximate composition, mineral and vitamin content, quantified the organic acids and phenolic compounds, and determined the antioxidant activity of three wild, edible mushrooms found in Manipur.

## 2. Results and Discussion

### 2.1. Nutritional Value

**Figure 1:**
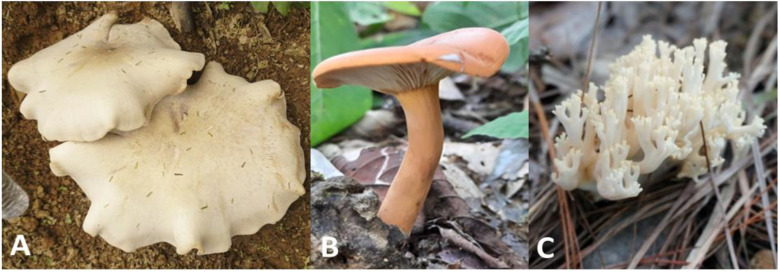
Fruiting body of the wild edible mushrooms in this study. (**A**) *Macrocybe gigantea* J124; (**B**) *Lactifluus leptomerus* J201; (**C**) *Ramaria thindii* J470.

The proximate compositions of the three wild edible mushrooms, *M. gigantea* J124, *L. leptomerus* J201 and *R. thindii* J470 are shown in Figure 1. The nutritional contents of the mushrooms were both species- and source-dependent and their ability to accumulate nutrients from the substrates. In the dried fruiting bodies, carbohydrate was the major constituent, followed by protein, ash, fiber and fat. Compared to the other mushroom species in this study, *M. gigantea* J124 had the highest protein, ash and moisture content, *L. leptomerus* J201 had the highest fiber and fat content, while *R. thindii* J470 had the highest carbohydrate content and energy value. The energy contribution of *R. thindii* J470 and *L. leptomerus* J201 was higher, because of the higher content of carbohydrate. The high ash content of *M. gigantea* J124 may contribute to its rich minerals and vitamins content. *L. leptomerus* J201 contains the highest percentage of fiber, supplementing the fiber intake in the diet, while *R. thindii* J470 has the highest carbohydrate and energy level, of 320 K cal. per 100 g dry weight. Overall, these results indicate that the mushrooms studied are good sources of proteins, crude fiber, carbohydrate, vitamins and minerals, though different nutritional composition richness varies by species.

Mushrooms are a rich source of quality proteins and these are reported to rank well in their quantity, above common vegetables and fruits [34]. The protein content of the species investigated was found to be 37.6%, 20.8% and 16.4%, corresponding to *M. gigantea* J124, *L. leptomerus* J201 and *R. thindii* J470, respectively. For *M. gigantea* J124, the protein content is 37.6%, which is slightly higher than previously studied [35], while the protein content is in correlation with another previously reported study for the same mushroom [13]. A previous report on the protein content of different *Ramaria* species ranged from 10.81 to 21.65%, which correlated with our finding of 16.4% for *R. thindii* J47 [17]. So far, there has been no report on the nutritional content of *L. leptomerus*; therefore, in this study, we report for the first time that *L. leptomerus* J201 contains an adequate value of 20.8% protein per dw. A previous study reported that another species of *Lactifluus, L. piperitus*, a species of the subgenus *Lactifluus* from Nagaland, contained 19.33% protein, which is almost similar to our study on the same subgenus [35].

As is evident from the nutritional profile of the different edible mushrooms in Table 1, carbohydrates constitute the largest fraction of mushrooms’ nutritional content per 100 g of dry matter. The results of the present study revealed that the carbohydrate content on a dry weight basis was highest in *R. thindii* J470 (57.2%), followed by *L. leptomerus* J201 (43%) and *M. gigantea* J124 (32%) In contrast, a previous report on the carbohydrate content of *M. gigantea* was 52.1%, which is higher than our findings [13], and another study reported 40 to 50% carbohydrate content in the Ramaria species, which is lower than *R. thindii* J470 (57.2%) [17]. Unlike the protein content, *M. gigantea* J124 has the lowest carbohydrate content percentage.

The crude fat content of the edible mushrooms has been reported to be less than 1% and as high as 15–20% [36]. The fat content percentage of the wild edible mushrooms for *R. thindii* J470, *M. gigantea* J124 and *L. leptomerus* J201 were 2.8%, 3.2% and 5.9%, respectively. The maximum percentage of crude fat was determined in *L. leptomerus* J201. It was previously reported that the fat content in the Ramaria species ranged from 0.22 to 1.49%, while in our study we reported a higher percentage in the case of *R. thindii* J470, which is 2.8% per dw [17]. The fat content percentage of *M. gigantea* J124 is 3.2%, which is higher than that previously reported [13].

Amongst the wild edible mushrooms evaluated in this study, *M. gigantea* J124 contains a maximum percentage ash content of 9.9% dry weight, which could directly contribute to its high bioaccumulation of minerals from its surroundings. A previous study reported ash contents of 4.53%, 6.23% and 7.36% in *M. gigantea*, which is lower than our present study [13,37]. In another study [17], the ash content of the Ramaria species ranged between 0.23 to 1.25%, which is relatively lower than our findings in the case of *R. thindii* J470 (7.9%). The minimum ash content was reported in *L. leptomerus* J201 (6.8%), which was slightly higher than previously reported in *L. piperatus* which belongs to the same subgenus [35].

Mushrooms are rich in fiber content. The fiber content percentage on a dry weight basis ranges from 5.9 to 14.1%. In contrary to its moisture, ash and protein content, a minimum fiber content of 5.9% was reported in *M. gigantea* J124, which is slightly higher than previously reported [13]. The fiber content of *R. thindii* J470 is 6.4%, which is substantially higher compared to that previously reported in the Ramaria species, which ranged between 0.28–1.33% [17]. The maximum fiber content was determined in *L. leptomerus* J201, with an elevated value of 14.1% in comparison with the other two edible mushrooms.

Moisture is an integral part of the proximate composition of mushrooms, as it gives an exact estimate of the various nutritional components. The exact value of the moisture content varies substantially from species to species, as is evident from the data given in Table 1. Crisan and Sands documented that an average mushroom contained approximately 90% moisture when fresh, and 10–12% when air-dried [36]. During the present study, the moisture content ranged from 83.846 to 89.44%. The maximum moisture content was detected in *M. gigantea* J124, which correlates with previously reported levels [37], but was slightly higher than the one reported by Roy Das et al. [13]. The minimum moisture content of 83.84% in *R. thindii* J470 and 84.79% in the case of *L. leptomerus* J201. Although moisture has no nutritional relevance, it does need to be taken into consideration to obtain the correct value of the important nutritional and nutraceutical constituents. As documented by Crisan and Sands, not evaluating the moisture percentage will normally result in inflated estimates of the various nutritional components present in the samples [36].

The maximum energetic values were observed in *R. thindii* J470, followed by *L. leptomerus* J201 and *M. gigantea* J124. The high energy value in *R. thindii* J470 relates to its high carbohydrate content. For *M. gigantea* J124, we reported 307 Kcal/100 g, which is lower than previously reported [13].

### 2.2. Vitamin Content

An important factor in the overall nutritional value of food is its vitamin content. Mushrooms are rich in vitamin B-complex, while poor in the fat-soluble vitamins (A, D, E and K) [16,21,38,39]. However, the vitamin contents were species dependent. During the present investigation, we present the first ever report on the vitamin content of the three edible wild mushrooms. The vitamin D, vitamin B1, B2, niacin, folic acid and vitamin C content were reported in these three wild edible mushrooms (Table 2). Amongst the evaluated vitamins, vitamin C was the most abundant vitamin detected, followed by niacin, vitamin D and vitamin B2, while vitamin B1 and folic acid are found below the detectable level. All of the three mushrooms contain equivalent amounts of vitamin D and vitamin C, while vitamin B2 is present in trace amounts. Vitamin C is a natural antioxidant while vitamin D and vitamin B (niacin) are essential for the proper distribution and absorption of other nutrients inside our body. *M. gigantea* J124 contains substantially higher niacin (51.5 mg/100 g) in comparison to the other two edible mushrooms (Table 2).

### 2.3. Mineral Content

Mushrooms are an excellent source of important minerals. About 56–70% of the total ash has been reported to be normally composed of different minerals, such as Ca, Mg, Na, K, P, etc. [40]. The mineral composition of mushrooms is reported to be largely influenced by the bioaccumulation of minerals through the growing mycelium from the substrate [41,42]. In most of the cases, K, Na and P were reported to be present in significant quantities, followed by Ca and Fe in low quantities [41]. The results of the present study to determine the proximate mineral content in the dried samples of wild edible species of *M. gigantea* J124, *L. leptomerus* J201 and *M. gigantea* J124 are summarized in Table 3. Minerals are micronutrients that are essential for the proper functioning of the body. Amongst the evaluated species, *M. gigantea* J124 possesses the highest concentration of most of the essential minerals (Ca, Cu, K, Mg, Na, P and Zn). Fe is present in an elevated concentration of 79.741 ppm in the mushroom *R. thindii* J470, as compared to *L. leptomerus* J201 and *M. gigantea* J124. Amongst the minerals analyzed, K was found to be the most abundant, followed by Fe, Na, Mg, Zn, Ca, Cu and Mn, while P was present in a minimal concentration in all of the three mushrooms. Most of the major minerals required for the body are present in all of the three wild edible mushrooms.

### 2.4. Organic Acids

The organic acids and phenolic acids content of the three mushrooms were identified by comparing their retention time with the standards, and the identified compounds are listed in Table 4. Oxalic acid, tartaric acid, formic acid and lactic acid were detected in all of the three mushrooms, while malic acid and malonic acid were present only in *M. gigantea* J124. Oxalic acid was present in a high concentration in all of the three mushrooms, with a concentration of 213 µg/mg, 38.985 µg/mg and 40.65 µg/mg in *M. gigantea* J124, *L. leptomerus* J201 and *R. thindii* J470, respectively (Table 4). In the case of *M. gigantea* J124, oxalic acid, tartaric acid, formic acid, malic acid and malonic acid were present in a substantially higher concentration in comparison to the other two species. Lactic acid was detected in a minimum concentration in the case of *M. gigantea* J124 while in *L. leptomerus* J201 and *R. thindii* J470, acetic acid and citric acid were detected at a minimum concentration, respectively. The organic acids, such oxalic acid and fumaric acid, are known to possess antibacterial, antioxidant and anti-inflammatory properties. Thus, the organic acids have considerable influence on the nutritional and nutraceutical potential of mushrooms.

### 2.5. Phenolic Compound

Gallic acids, hydroxybenzoic acid and quercetin are present in all three mushrooms and epicatechin is present only in the case of *R. thindii* J470. The maximum phenolic compounds were detected in *R. thindii* J470, which correlates with the presence of a high total phenolic content and its radical scavenging properties, as compared to the other two mushrooms. Hydroxybenzoic acid was the most abundant phenolic compound present in *M. gigantea* J124 and *R. thindii* J470, while gallic acid was the most abundant in case of *L. leptomerus* J201. Epicatechin was detected only in *R. thindii* J470, at a concentration of 602.201 µg/g.

### 2.6. Antioxidants

#### DPPH Scavenging Activity

**Figure 2:**
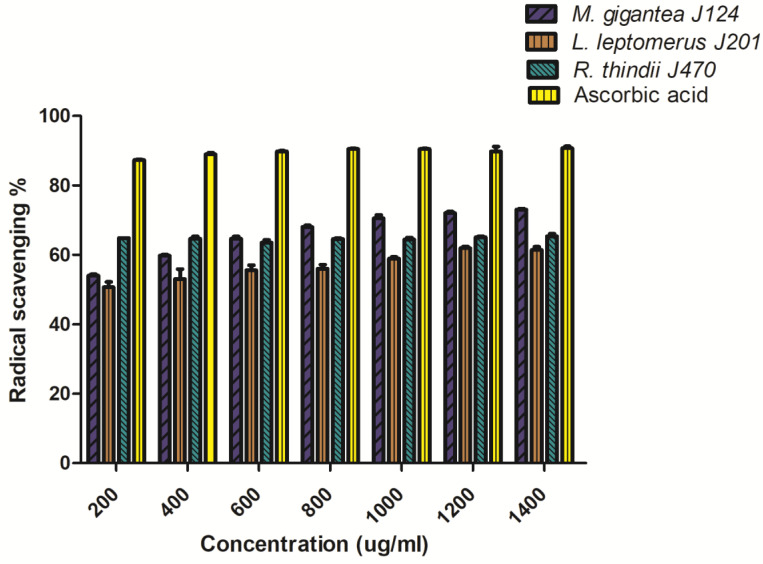
Comparative results of the DPPH radical scavenging percentage of methanol extract of the three wild edible mushrooms and ascorbic acid at different concentrations (mean ± SEM).

The antioxidant capacity of the methanolic extracts against oxidation caused by free radicals was determined by DPPH scavenging activity, which shows that *M. gigantea* J124 and *R. thindii* J470 exhibit a higher capacity of scavenging by the DPPH radical than *L. leptomerus* J201 (Figure 2). Ascorbic acid was used as a positive control, which exhibits a DPPH radical scavenging activity of 90.722% at 1400 µg/mL and an IC50 value of 428.0 µg/L. The IC50 value for *R. thindii* J470, *M. gigantea* J124 and *L. leptomerus* J201 is 242.0 µg/mL, 550.4 µg/mL and 689.0 µg/mL, respectively. Significant differences (*p* < 0.0001) were observed between the mushrooms studied. The maximum radical scavenging activity percentages of 73.1%, 65.37% and 61.43% were observed at 1400 µg/mL concentration of the *M. gigantea* J124, *R. thindii* J470 and *L. leptomerus* J201 extracts, respectively.

The graphical presentation of radical scavenging activity was plotted against extract concentration (Figure 2). The mushroom *R. thindii* J470 extract exhibited the highest radical scavenging percentage, even at the lowest concentration, which concludes that the higher concentration of phenolic compounds in *R. thindii* J470 contributes to its radical scavenging properties. This relationship was proven by the Pearson correlation analysis between DPPH radical scavenging activity, total phenolic content and total flavonoid content and it was observed that the DPPH radical scavenging activity and total phenolic content value are significant with *p* value (two-tailed) of 0.0396 and an R^2^ value of 0.9961, while the correlation between DPPH scavenging activity with total flavonoid content was not significant. The antioxidant components and the correlation with IC50 values were visible in the wild edible mushrooms that were studied. The main effect of the phenolic compounds is the radical scavenging effect.

## 3. Materials and Methods

### 3.1. Collection, Storage and Samples Preparation

**Figure 3:**
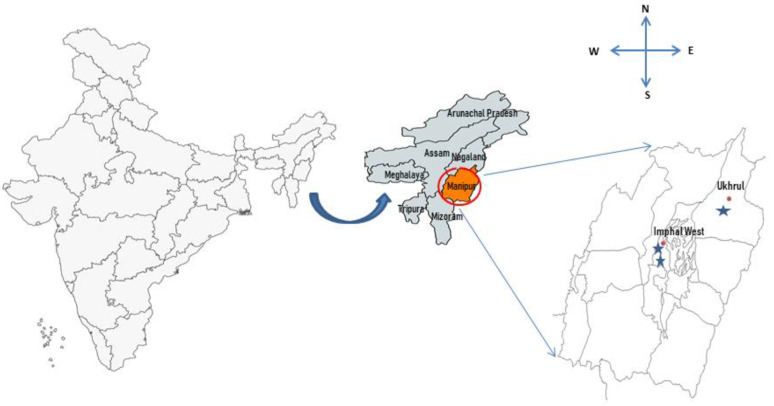
Collection sites of the wild edible mushrooms in Manipur.

The mature fruiting bodies of the wild edible mushrooms were collected from different districts of Manipur (Figure 3). Each fruiting body was handpicked from their natural habitats and cleaned from soil substrates. The wild mushrooms were identified by microscopic and macro-morphological examination and the identification of each sample was authenticated by referring to several authors, *Macrocybe gigantea* [22], *Lactifluus leptomerus* [43] and *Ramaria thindii* [44]. *L. leptomerus* J201 and *R. thindii* J470 were collected from the hills of the reserve forest area near the National Game Village, Imphal West and *M. gigantea* J124 was collected from a reserve forest area situated near to Shirarakhong village of Ukhrul District, respectively, during June–October. All of the samples were then dried at 60 °C for 24 h to obtain a constant weight. The dried mushroom samples were then ground into a fine powder and stored at 4 °C for further analysis. A total of 10 g each of grounded *M. gigantea* J124, *L. leptomerus* J201 and *R. thindii J470* were stirred with methanol (100 mL) at 150 rpm for 24 h, and filtered through Whatman no. 4 paper. The residues were further extracted with an additional portion of methanol [45]. The methanolic extracts were evaporated using a rotary evaporator under reduced pressure, re-dissolved in dimethyl sulfoxide (DMSO) at a concentration of 50 mg mL^−1^ (stock solution) and stored at 4 °C for further analyses of its antioxidant assays.

### 3.2. Nutritional Value

The nutritional compositions of the mushrooms were expressed on percentage dry weight basis. The content of moisture, proteins, fat, ash, fiber, carbohydrate and the energy value were determined according to the Association of Official Agricultural Chemists (AOAC) (2016) procedure [46]. For the estimation of the crude protein content (N × 4.38) the macro-Kjeldahl method was used, the crude fat content was determined by extracting a known weight of sample with petroleum ether using a Soxhlet apparatus, while the ash content was determined by calcination at 600 ± 15 °C. The carbohydrate content was calculated from the sum of the percentages of crude protein, ash, fat and crude fiber subtracted from 100 and the total energy was calculated according to the following equation:

Energy (kcal) = 4 × (g protein + g carbohydrates) + 9 × (g fat)(1)

### 3.3. Vitamin Content

The different vitamin contents of the three mushrooms were analyzed by the following standard procedures, according to the Association of Official Agricultural Chemists (AOAC) (2016) [46]: Vitamin D (995.05, 2002.05); Vitamin B1 (957.17); Vitamin B2 (970.65); Niacin (961.14); Folic acid (2011.06) and Vitamin C (967.21) [46] at the Department of Food Safety and Analytical Quality Control Laboratory, CSIR–Central Food Technological Research Institute, Mysuru.

### 3.4. Mineral Analysis

The analysis for Sodium (Na), Calcium (Ca), Potassium (K), Phosphorus (P), Magnesium (Mg), Chromium (Cr), Manganese (Mn), Iron (Fe), Cobalt (Co), Copper (Cu), Zinc (Zn), Selenium (Se), Molybdenum (Mo), Lead (Pb), Cadmium (Cd), Boron (B) and Barium (Ba) was carried out using (ICP-OES) Inductively Coupled Plasma-Optical Emission Spectrometry (ICP-OES), Thermo Scientific^TM^ iCAP^TM^ 7600, Waltham, MA, USA [47], using the following parameters.

Method parameters.

**Table:** 

View Direction	Radial	Axial
UV exposure time	15	15
UV RF Power	1150	1150
UV Neb Gas Flow	0.5	0.5
VIS Exposure Time	5	5
VIS RF Power	1150	1150
VIS Neb Gas Flow	0.5	0.5
Cool Gas Flow Rate	12	12
Aux Gas Flow Rate	0.5	0.5

### 3.5. Phenolic Compounds by HPLC

The standard chemicals were purchased from Sigma-Aldrich Co. (St. Louis, MO, USA), HPLC-grade methanol (RANKEM, AVANTOR, Gujarat, India), acetic acid (Merck Worli, Mumbai, India) and Milli-Q water (Merck Millipore, Kolkata India. For the preparation of the stock solution ascorbic acid, (1 mg/mL) was dissolved in 0.5 mL methanol followed by sonication for 10 min and the resulting volume was made up to 1 mL with the solvent for the mobile phase (acetonitrile and 1% aq. acetic acid 1:9). To prepare the stock standard solutions of the phenolic acids, the same method was followed. The working solutions of the sample were prepared by further dilution of the standard solution with the mobile phase solvent system. The working solutions and standards were filtered through 0.45 μm syringe filter (Moxcare Labware, Haryana, India) and the mobile phase was degassed before the injection of the solutions [48].

The HPLC analyses was performed by CBM-20A, LC-20AD prominence Liquid chromatography, Shimadzu SPD-M20 A DAD, UFCL, with a two solvent delivery system using binary pump (LC-20AD), including a diode array detector (SPD-M20A) with 2.5 cm flow cell, a manual sample injection valve equipped with a 20 μL loop and Shimadzu LC solution version 1.25 system manager as the data processor. The separation was achieved by an analytical Zorbak SB-C18 column (4.6 mm × 250 mm × 5 µm), SN- USCLO66936, L. N: B16111, Agilent Technologies, Santa Clara, CA, USA [48].

### 3.6. Organic Acid by HPLC

A HPLC system (Shimadzu, Tokyo) with a CBM-20A, LC-20ADprominence Liquid chromatography, Shimadzu SPD-M20 A DAD, UFCL, equipped with an analytical Zorbak SB-C18 column (4.6 mm × 250 mm × 5 µm), SN- USCLO66936, L. N: B16111, Agilent Technologies, maintained at 30 °C, was used for the analysis of the organic acids.

All of the samples were filtered in triplicate through a 0.22 mm filter unit (Moxcare Labware, Haryana, India) before injection and the solvents were filtered through a 0.45 mm filter (Moxcare Labware, Haryana, India). A solvent system, comprising Milli-Q water: phosphoric acid (99.9:0.1), was used as a mobile phase at a flow rate of 1 mL/min and the injection volume was 20 mL. The run time was 15 min and the detection of organic acids was carried out at 230 nm [49].

For identification, the retention times of the peaks of the investigated compounds were compared with standard peaks in the same conditions, as well as by spiking the samples with stock standard solutions. The concentrations of the identified compounds in the extract were calculated with the regression parameters obtained from calibration curves. All of the standard calibration curves showed high degrees of linearity (r2 > 0.99). The standard organic acids used were oxalic acid, tartaric acid, formic acid, malic acid, malonic acid, lactic acid, acetic acid and citric acid (Sigma Aldrich, Spruce Street, St. Louis, MO, USA).

### 3.7. Total Phenolic Content

The total phenol contents in the extracts were determined, using a slight modification of the Folin–Ciocalteu method [50,51,52,53]. A total of 50 µL aliquots of 12.5, 25, 50, 100, 200 and 400 µg/mL methanolic gallic acid solutions were mixed with 100 µL Folin–Ciocalteu reagent (diluted tenfold) and 100 µL (75 g/L) sodium carbonate. The mixture was incubated at 25 °C for 30 min; the quantitative phenolic estimation was performed at 765 nm and the calibration curve was constructed by plotting the absorbance against the concentration. A similar procedure was conducted for the test samples, as described above. The total phenolic content was expressed as micrograms of gallic acid equivalent (GAE) per gram of extract using the following equation based on the calibration curve: y = 0.0032x + 2.1684, R² = 0.9831, where “x” was the absorbance and “y” was the gallic acid equivalent (mg/g).

### 3.8. Total Flavonoid Content

The flavonoid content was determined by following the protocol of Herald TJ et al. (2012) [51]. Distilled water (100 μL) was added to each of the 96 wells, followed by 10 μL of 50 g/L NaNO_2_ and 25 μL of standard or sample solution. After 5 min, 15 μL of 100 g/L AlCl3 was added to the mixture; 6 min later, 50 μL of 1 mol/L NaOH and 50 μL of distilled water was added. The plate was shaken for 30 s in the plate reader prior to absorbance measurement at 510 nm. The total flavonoid content was calculated as quercetin (mg/g) using the following equation based on the calibration curve: y = 0.0002x + 0.0271, R² = 0.9871, where “x” was the absorbance and “y” was the quercetin equivalent (mg/g).

### 3.9. Antioxidants

#### DPPH Assay

The DPPH free radical scavenging activities were conducted by the modification method of Blois (1958) in 96-well microplates [52,54]. The crude product of the three mushroom extracts was diluted to concentrations of 1400, 1200, 1000, 800, 600, 400 and 200 (µg/mL) in DMSO. 90 µL of 0.1 mM DPPH (dissolved in MeOH) added to 90 µL of sample solutions of different concentrations to make up the total volume of 180 µL. The test reactions were mixed thoroughly in the 96-well plates, incubated at 37 °C for 30 min, and absorption was measured at 516 nm with a UV spectrophotometer. The extract concentration providing 50% inhibition (IC50) was calculated for the scavenging activity comparison. Ascorbic acid was used as positive control. The percentage of DPPH free radical scavenging activity was calculated as follows:

RSA on DPPH (%) = [(A control − A sample)/(A control)] × 100(2)

where:

A sample = the absorbance of the extract/reference;
A control = the absorbance of the DPPH solution without the addition of extract.

### 3.10. Statistical Analysis

Statistical analyses were conducted using GraphPad Prism version 5 for Windows. All of the determinations were carried out in triplicate and the data were presented as mean ± standard error mean.

## 4. Conclusions

In the current study, we provide the nutritional and bioactive profile of the three wild edible mushrooms, such as proteins, carbohydrate, fats, ash, vitamins, minerals, organic acids, phenolic compounds and their antioxidant potential. The experimental results reveal that all of the three wild edible mushrooms are an excellent source of proteins, carbohydrate, vitamin C and other essential minerals. In terms of the content of proteins, ash, minerals and vitamins, *M. gigantea* J124 outperformed the other mushrooms, however, *R. thindii* J470 exhibited the highest total phenolic and flavonoid content, which corresponded to its highest radical scavenging properties. Vitamin C and vitamin D were present in an almost equivalent percentage in all of the three mushrooms. The presence of vitamins and minerals within the human diet is incredibly important. In addition, extensive explorations and thorough screening of the nutritional value of wild edible mushrooms is essential, as mushrooms are a valuable natural resource to mankind with immense health benefits. Since these mushrooms are rarely found, the development of mass production technology is highly recommended. The knowledge and documentation of the baseline vitamin and mineral composition of the wild-growing mushrooms are essential to take care of nutritional needs, especially for those people who are vegetarian or maintain a vegan diet.

## Figures and Tables

**Table 1:** Nutritional value of the three edible wild mushrooms in dry weight.

Samples	Moisture (%)	Ash (%)	Fat (%)	Fiber (%)	Protein (%)	Carbohydrates (%)	Energy Value Kcal/100 g
*M. gigantea* J124	89.44 ± 0.16	9.90 ± 0.04	3.20 ± 0.04	5.90 ± 0.04	37.60 ± 0.09	32.00 ± 0.40	307 ± 0.40
*L. leptomerus* J201	84.79 ± 0.22	6.80 ± 0.05	5.90 ± 0.05	14.10 ± 0.05	20.80 ± 0.05	43.00 ± 0.57	308 ± 0.57
*R. thindii* J470	83.84 ± 0.26	7.90 ± 0.05	2.80 ± 0.05	6.40 ± 0.05	16.40 ± 0.05	57.20 ± 0.05	320 ± 0.57

Each Value is expressed in mean ± SEM, (*n* = 3) dry weight.

**Table 2:** Vitamin content of the three wild edible mushrooms mg/100 g dry weight.

Mushroom	Vitamin D (µg/g)	Vitamin B1 (mg/100 g)	Vitamin B2 (mg/100 g)	Niacin (mg/100 g)	Folic Acid (mg/100 g)	Vitamin C (mg/100 g)
*M. gigantea* J124	2.85 ± 0.009	BDL	0.38 ± 0.009	51.50 ± 0.090	BDL	33.00 ± 0.400
*L. leptomerus* J201	2.87 ± 0.057	BDL	0.36 ± 0.005	7.70 ± 0.057	BDL	34.00 ± 0.570
*R. thindii* J470	2.83 ± 0.005	BDL	0.40 ± 0.005	7.30 ± 0.057	BDL	32.00 ± 0.288

Results are presented as mean ± SEM, (*n* = 3); BDL stands for below detectable limit. All vitamins are presented in mg/100 g dry weight except for vitamin D (µg/g).

**Table 3:** Mineral content of edible mushrooms in dry weight.

Minerals	*M. gigantea* J124	*L. leptomerus* J201	*R. thindii* J470
Ca	2.778 ± 0.153	1.699 ± 0.003	1.237 ± 0.111
Cu	5.771 ± 0.020	0.368 ± 0.115	0.149 ± 0.018
Fe	7.467 ± 0.199	6.645 ± 0.113	79.741 ± 0.194
K	210.380 ± 0.215	128.35 ± 0.200	133.99 ± 0.199
Mg	4.638 ± 0.064	1.663 ± 0.182	2.924 ± 0.123
Mn	0.225 ± 0.016	0.244 ± 0.001	1.023 ± 0.037
Na	4.488 ± 0.055	2.901 ± 0.085	2.827 ± 0.018
P	0.016 ± 0.0005	n.d.	0.004 ± 0.0005
Zn	4.149 ± 0.036	1.097 ± 0.041	1.252 ± 0.077

Results are presented as mean ± SEM (*n* = 3). Each value is expressed in ppm; n.d.—not detected.

**Table 4:** Organic acid and phenolic compound, total phenolic, total flavonoid and DPPH IC50 of the three wild edible mushrooms.

Peak No.	Compounds	*M. gigantea* J124	*L. leptomerus* J201	*R. thindii* J470
1	Organic acid (µg/mg) Oxalic acid	213 ± 0.0009	38.985 ± 0.001	40.65 ± 0.0003
2	Tartaric acid	134.567 ± 0.00028	33.713 ± 0.001	37.217 ± 0.001
3	Formic acid	50.662 ± 0.00039	33.047 ± 0.0018	27.88 ± 0.0022
4	Malic acid	9.7675 ± 0.00021	n.d.	n.d.
5	Malonic acid	7.665 ± 0.00031	n.d.	n.d.
6	Lactic acid	6.77 ± 0.004	7.437 ± 0.001	9.255 ± 0.0005
7	Acetic acid	n.d.	2.685 ± 0.0019	1.649 ± 0.0004
8	Citric acid	n.d.	4.072 ± 0.0009	0.444 ± 0.0023
1	Phenolic Compound (µg/g) Gallic acid	51.399 ± 0.01	168.542 ± 0.01	253.827 ± 0.07
2	Hydroxybenzoic acid	585.56 ± 0.022	31.285 ± 0.016	1075.47 ± 0.014
3	Epicatechin	n.d.	n.d.	602.201 ± 0.011
4	Quercetin	94.699 ± 0.007	11.271 ± 0.019	92.127 ± 0.007
	TPC mg GAE/g dw	29.23 ± 0.09	26.206 ± 0.077	30.99 ± 0.27
	TFC mg QE/g dw	6.646 ± 0.452	6.854 ± 0.517	9.187 ± 0.511
	DPPH IC50 (µg/mL)	550.4	689.0	242.0

Results are presented as mean ± SEM (*n* = 3); n.d.—Not detected; TPC stands for total phenolic content; TFC stands for total flavonoid content; GAE stands for gallic acid equivalents, QE stands for quercetin equivalents, DPPHIC50 is the concentration of the sample that can scavenge 50% of DPPH free radical, dw stands for dry weight.

## Data Availability

Not applicable.

## Sample Availability

Samples are available from the authors.
